# Anaerobic Process for Bioenergy Recovery From Dairy Waste: Meta-Analysis and Enumeration of Microbial Community Related to Intermediates Production

**DOI:** 10.3389/fmicb.2018.03229

**Published:** 2019-01-08

**Authors:** Giorgia Pagliano, Valeria Ventorino, Antonio Panico, Ida Romano, Francesco Pirozzi, Olimpia Pepe

**Affiliations:** ^1^Department of Agricultural Sciences, University of Naples Federico II, Naples, Italy; ^2^Task Force on Microbiome Studies, University of Naples Federico II, Naples, Italy; ^3^Telematic University Pegaso, Naples, Italy; ^4^Department of Civil, Architectural and Environmental Engineering, University of Naples Federico II, Naples, Italy

**Keywords:** microbiota, dairy by-products valorization, anaerobic biosystem, *Methanoculleus*, hydrogen, methane

## Abstract

Dairy wastes are widely studied for the hydrogen and methane production, otherwise the changes in microbial communities related to intermediate valuable products was not deeply investigated. Culture independent techniques are useful tools for exploring microbial communities in engineered system having new insights into their structure and function as well as potential industrial application. The deep knowledge of the microbiota involved in the anaerobic process of specific waste and by-products represents an essential step to better understand the entire process and the relation of each microbial population with biochemical intermediates and final products. Therefore, this study investigated the microbial communities involved in the laboratory-scale anaerobic digestion of a mixture of mozzarella cheese whey and buttermilk amended with 5% w/v of industrial animal manure pellets. Culture-independent methods by employing high-throughput sequencing and microbial enumerations highlighted that lactic acid bacteria, such as *Lactobacillaceae* and *Streptococcaceae* dominated the beginning of the process until about day 14 when a relevant increase in hydrogen production (more than 10 ml H_2_ gVS^-1^ from days 13 to 14) was observed. Furthermore, during incubation a gradual decrease of lactic acid bacteria was detected with a simultaneous increase of *Clostridia*, such as *Clostridiaceae* and *Tissierellaceae* families. Moreover, archaeal populations in the biosystem were strongly related to inoculum since the non-inoculated samples of the dairy waste mixture had a relative abundance of archaea less than 0.1%; whereas, in the inoculated samples of the same mixture several archaeal genera were identified. Among methanogenic archaea, *Methanoculleus* was the dominant genus during all the process especially when the methane production occurred, and its relative abundance increased up to 99% at the end of the incubation time highlighting that methane was formed from dairy wastes primarily by the hydrogenotrophic pathway in the reactors.

## Introduction

In the near future, novel bio-based technologies in waste management can be used to convert organic waste into valuable products such as renewable energy and/or biopolymers through biological processes (Pagliano et al., 2017) with a goal to potentially replace fossil fuels with biomasses and reducing pollutant emissions.

Several organic wastes are potentially suitable to be used as substrates for producing renewable energy vectors (e.g., biohydrogen, biogas, and biomethane) through anaerobic biosystems (Raposo et al., 2012; Ghimire et al., 2015; Pagliano et al., 2017). Among them, cheese whey and buttermilk, residues from dairy factories as by-products of cheese, yogurt, milk, and butter production process are interesting substrates for their high content of soluble organic matter, i.e., chemical oxygen demand (COD) ranging from 0.1 to 100 g L^-1^ (Prazeres et al., 2012). Besides substrates and operational conditions, microorganisms significantly affect the performance of the anaerobic process (Panico et al., 2014). In fact, the efficiency and stability of this process is entirely dependent upon the syntrophic activity of microorganisms operating in different phases (Li et al., 2009; Vanwonterghem et al., 2014).

Actually, anaerobic digestion process can be conceptually divided into four stages defined by the primary catabolic reactions that occur at each stage: hydrolysis of complex polymers (I, hydrolysis), fermentation of the hydrolysis end-products to volatile fatty acids (VFAs) (II, acidogenesis), conversion of VFAs to acetate and hydrogen (III, acetogenesis), and finally the production of methane from acetate and hydrogen (IV, methanogenesis) (Yu et al., 2010). Therefore, it is important to understand how the raw materials as well as environmental and physical conditions established in the system affect microbial growth and activity, and therefore, the performance of the anaerobic process. Numerous studies using different types of organic wastes have been conducted to better understand the role of the microorganisms involved in each stage and the microbiomes present in the anaerobic reactors (Nelson et al., 2011; Li et al., 2017; Ros et al., 2017; Westerholm et al., 2017). For this purpose, various methods have been applied to investigate the microbial communities or targeted specific groups in anaerobic digesters, including clone library of 16S rRNA genes (Rincón et al., 2008), denaturing gradient gel electrophoresis (DGGE) analysis (Shin et al., 2010; Palatsi et al., 2011; Supaphol et al., 2011; Pagliano et al., 2018; Ventorino et al., 2018) and fluorescence *in situ* hybridization (FISH) (Braguglia et al., 2012). All these methods, although are highly efficient, analyze a limited number of aspects if compared with the emerging metagenomic approaches based on high-throughput sequencing (HTS) (Yang et al., 2014). Therefore, in this study, the use of a polyphasic approach including HTS in lab-scale batch tests, allowed to elucidate the dynamics of microbiota in different stages of the anaerobic process fed with a mixture of dairy waste from a mozzarella cheese factory. Culture-independent and culture-dependent approaches coupled with hydrogen and methane production can improve the knowledge concerning this specific anaerobic biosystem. In particular, it is important to elucidate how specific microbial populations can steer the hydrogen and methane production in order to control, also through traditional parameters (pH, TS, VS, COD) (Pontoni et al., 2015), the efficiency of the anaerobic biosystems fed with different wastes and by-products from dairy industry.

## Materials and Methods

### Dairy Wastes Characterization and Experimental Set Up

Thirty L samples of cheese whey and buttermilk were collected from the production chain of buffalo mozzarella cheese operating at a temperature of approximately 33–37°C and immediately analyzed for physical–chemical (pH, TTA, TS, VS, COD) and microbial characterization. The pH was measured using a HI 221 pH meter (Hanna Instruments, Inc., Woonsocket, RI, United States). Total titratable acidity (TTA) was calculated as the mL of 0.1N NaOH 10 mL^-1^ of sample (AACC, 1975). Total solids (TS) and volatile solids (VS) were evaluated as described in the standard methods (APHA, 2005). COD was measured with an ECO08 thermoreactor (VELP Scientifica, Usmate Velate, Italy) and a PF-3 photometer (VELP Scientifica) using kit NANOCOLOR^®^.

Microbiological counts were performed on serially diluted cheese whey, buttermilk, and the mixture of them, which were spread on the plate surface containing different media. Heterotrophic aerobic and anaerobic bacteria were counted on Plate Count Agar (Oxoid, Milan, Italy) and incubated for 48 h at 30°C under either aerobic or anaerobic conditions (Oxoid’s Anaerogen^TM^ System, Oxoid). Spore-forming bacteria were cultivated on Plate Count Agar (Oxoid) after a pretreatment at 80°C for 10 min, and the plates were incubated at 30°C for 48 h in aerobic or anaerobic conditions. Jactic acid bacteria (LAB) were counted on MRS agar (Oxoid), and the plates were incubated for 48 h at 30°C. *Clostridia* were enumerated on Reinforced Clostridial Medium (Oxoid), and the plates were incubated for 48 h at 30°C under anaerobic conditions. Enterococci were counted on the selective substrate, Slanetz-Bartley agar (Oxoid), after incubation at 37°C for 48 h.

For the experimental plan, 6 L steel vessels were used as anaerobic biodigesters (working volume of 5 L). Each biodigester was equipped with a manometer and valves for biogas collection as well as effluent discharge.

Biodigesters were filled with a mixture of cheese whey and buttermilk (ratio 2:1 v/v, respectively, to simulate the characteristics of a real dairy waste stream produced from a mozzarella cheese factory) and inoculated with 5% (w/v) of industrial animal manure pellets (Stalfert N2 – Organazoto Fertilizzanti s.p.a, Pistoia, Italy). Tests were performed in duplicate (B1 and B2) at 30 ± 1°C and for 30 days.

### Anaerobic Biosystem Monitoring

#### Biological Gas Production and Intermediate Products Evaluation

Biological gas production was measured with a volumetric displacement method (Esposito et al., 2012) using teflon bags (maximum capacity of 10 L) to collect and storage biogas until analyses. The volume of biogas produced was measured by connecting each teflon bag with a capillary tube to an inverted 1000 mL glass bottle containing an acid solution (1.5% HCl) (Ghimire et al., 2015). Gas was detected using a Varian Star 3400 gas chromatograph (Agilent, Santa Clara, CA, United States) equipped with a Shin Carbon ST 80/100 column and a thermal conductivity detector. Argon was used as the carrier gas with an operating pressure of 20 psi. Gas measurements were performed daily during the first week and every 3 days during the following 3 weeks of incubation.

At 0, 7, 14, 21 and 30 days, liquid samples were collected from the biological reactors to analyze the concentration of lactose, galactose, lactic acid, acetic acid, propionic acid, and ethanol by high-performance liquid chromatography (HPLC, refractive index detector 133; Gilson system; pump 307, column Metacarb 67 h from Varian with 0.4 mL min^-1^ flow of 0.01 N H_2_SO). The pH and TTA were also constantly monitored.

#### Monitoring of Microbial Growth by Cultural Dependent Analysis

Liquid samples were collected from the biological reactors every week during 30 days of incubation. Bacterial counts were performed using either generic or selective differential growth media and, in particular, representative samples were characterized for heterotrophic aerobic and anaerobic bacteria, aerobic and anaerobic spore-forming bacteria, LAB, *Clostridia* and enterococci, as above described.

A one-way ANOVA followed by a Tukey test for pairwise comparison of means (*p* ≤ 0.05) were used to assess the difference in microbial counts at different incubation times. Statistical analyses were performed using the SPSS 21.0 statistical software package (SPSS, Inc., Cary, NC, United States) as reported by Ventorino et al. (2016).

#### Microbiota Analysis by High-Throughput Sequencing of the 16S rRNA Gene

Total genomic DNA was extracted from the liquid samples collected from the biodigesters using a FastDNA SPIN Kit for Soil (MP Biomedicals, Illkirch-Graffenstaden, France) according to the manufacturer’s instructions.

The microbial diversity was evaluated by amplicon based metagenomics sequencing using the primers S-D-Bact-0341F50 (5′-CCTACGGGNGGCWGCAG-3′) and S-D-Bact-0785R50 (5′-GACTACHVGGGTATCTAATCC-3′) (Klindworth et al., 2013) for bacterial communities; while for archaea, two different PCR reactions were performed. For the first round, the universal synthetic oligonucleotide primers, Arch 46f (5′-YTA AGC CAT GCR AGT-3′) (Øvreas et al., 1997) and Arch 1017r (5′-GGC CAT GCA CCW CCT CTC-3′) (Barns et al., 1994), were used as previously reported (Ventorino et al., 2018). For the second round, a nested PCR was performed using the primers Arch516F (5′-TGYCAGCCGCCGCGGTAAHACCVGC-3′) and Arch915R (5′-GTGCTCCCCCGCCAATTCCT-3′) (Raymann et al., 2017). Amplicon purification, multiplexing, and sequencing were carried out by Genomix4Life s.r.l. (Salerno, Italy) as reported in the Illumina 16S Metagenomic Sequencing Library Preparation manual. Sequencing was carried out on a MiSeq platform (Illumina Italy s.r.l., Milan, Italy), leading to 250 bp, two paired-end reads.

### Bioinformatics and Data Analysis

Row reads were qualitatively analyzed and filtered using PRINSEQ (Schmieder and Edwards, 2011). Low quality reads (Phred score < 20) were trimmed and reads shorter than 60 bp were discarded in end-to-end, sensitive mode. Paired-ends reads were merged using FLASH (Magoè and Salzberg, 2011) and sequences were then analyzed using QIIME 1.9.1 software (Caporaso et al., 2010). Operational taxonomy units (OTUs) at 97% sequence identity were picked through a *de novo* approach and the UCLUST method and taxonomic assignment was obtained using the RDP classifier and the Greengenes (McDonald et al., 2012). To avoid biases due to different sequencing depths, OTU tables were rarefied at the lowest number of sequences per sample.

Alpha diversity was evaluated by rarefaction curves, Good’s coverage, and Shannon diversity index (Shannon and Weaver, 1949). Beta diversity was also evaluated by UniFrac (Lozupone et al., 2006), and PCoA was generated by QIIME. To test the significant differences, statistical analyses were performed as previously described (Ventorino et al., 2015).

Phylogenetic trees for the representative bacterial and archaeal OTUs detected in this study and sequences downloaded from NCBI were constructed in Mega 4 by the Neighbor-Joining method using a maximum composite likelihood model with 1,000 bootstrap replicates.

### Data Accessibility

The raw Illumina sequencing data are available in the Sequence Read Archive database of the National Center of Biotechnology Information (SRP155825).

## Results

### Characterization of Dairy Wastes

**FIGURE 1:**
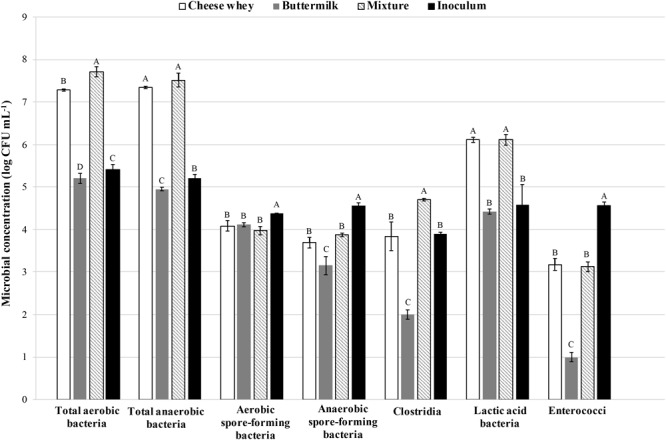
Viable counts of main bacterial groups evaluated in samples of cheese whey, buttermilk, and their mixture. The error bars represent the means ± SD of two replicates. Different letters indicate significant differences (p < 0.05).

Viable counts of main bacterial groups were evaluated in samples of cheese whey, buttermilk, and their mixture (Figure 1). The results showed a microbial load higher in cheese whey than in buttermilk, detected almost for each principal microbial group investigated. Actually, heterotrophic aerobic and anaerobic bacteria as well as LAB showed a higher concentration in the cheese whey (7.28 ± 0.02, 7.34 ± 0.02, and 6.11 ± 0.01 log CFU mL^-1^, respectively) than in the buttermilk (5.20 ± 0.05, 4.95 ± 0.04, and 4.42 ± 0.06 log CFU mL^-1^, respectively). Aerobic and anaerobic spore-forming bacteria were 4.08 ± 0.12 and 3.69 ± 0.12 log CFU mL^-1^ as well as 4.11 ± 0.05 and 3.15 ± 0.21 log CFU mL^-1^, respectively, for cheese whey and buttermilk. Moreover, the *Clostridia* load in cheese whey (3.84 ± 0.33 log CFU mL^-1^) was approximately two orders of magnitude greater than that recovered in buttermilk (2.02 ± 0.10 log CFU mL^-1^).

The inoculum showed a heterotrophic aerobic and anaerobic bacteria load of 5.41 ± 0.12 and 5.20 ± 0.90 log CFU mL^-1^, respectively, whereas, loads of aerobic and anaerobic spore-forming bacteria and LAB were around 4.5 log CFU mL^-1^.

Enterococci were detected in higher amount in inoculum (4.56 log CFU mL^-1^) and cheese whey (3.17 log CFU mL^-1^) samples.

Chemical characteristics of substrates and inoculum are listed in Table 1. The COD value was higher in cheese whey (74.10 ± 0.40 g L^-1^) than buttermilk (14.57 ± 0.20 g L^-1^) and the resulting mixture had a COD value closer to cheese whey than buttermilk (55.45 ± 0.22 g L^-1^). Obviously, TS and VS concentrations of cheese whey influenced the mixture concentrations resulting in 37.60 ± 0.01 and 34.53 ± 0.01 g L^-1^, respectively. The pH measurement did not drop below 5.0 for cheese whey, buttermilk and their mixture, while the inoculum had a value of 7.8 (Table 1); the TTA was 0.80 ± 0.10 °SH in the cheese whey as well as in the mixture but was lower in the buttermilk (0.40 ± 0.10 °SH).

**Table 1:** Physico-chemical characterization of cheese whey, buttermilk, their mixture and the animal manure inoculum.

Samples	COD (g L^-1^)	TS (g L^-1^)	VS (g L^-1^)	pH	TTA (°SH)
Cheese whey (W)	74.10 ± 0.40	54.34 ± 0.02	49.37 ± 0.01	5.00 ± 0.10	0.80 ± 0.10
Buttermilk (B)	14.57 ± 0.20	12.07 ± 0.01	9.91 ± 0.01	5.00 ± 0.10	0.40 ± 0.10
Mix of W and B^∗∗^	55.45 ± 0.22	37.60 ± 0.01	34.53 ± 0.01	5.00 ± 0.10	0.80 ± 0.10
Inoculum^∗∗∗^	n.d.^∗^	0.95 ± 0.0	0.43 ± 0.0	7.80 ± 0.10	n.d.^∗^

*^∗^Not determined; ^∗∗^(ratio 2:1 v/v); ^∗∗∗^animal manure.*

### Biological Gas Production and Biosystem Monitoring

**FIGURE 2:**
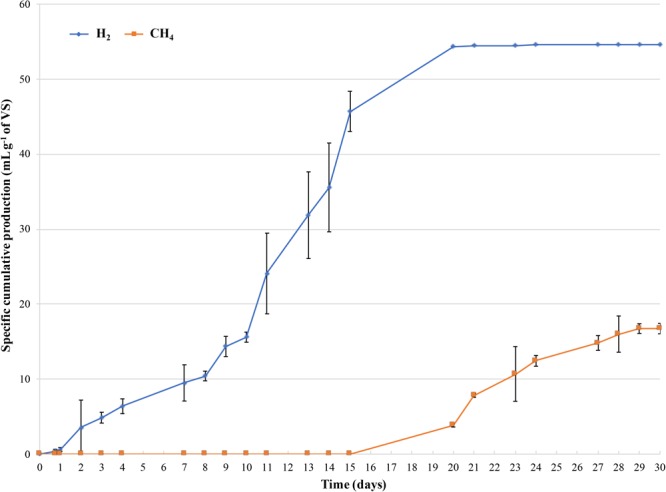
Cumulative specific production of H_2_ and CH_4_ monitored during 30 days of incubation. X-axis displays the day, and the Y-axis is the corresponding cumulative H_2_ or CH_4_ specific production per gram of the initial concentration of VS of the dairy waste mixture. The error bars represent the means ± SD of two replicates.

Figure 2 shows the cumulative volumes of the specific production of H_2_ and CH_4_ during incubation. After 7 days of incubation, a H_2_ production of 9.49 ± 2.41 ml H_2_ g^-1^ of VS was observed, increasing up to 54.35 ± 0.15 ml H_2_ g^-1^ of VS after 20 days. Regarding to biogas production, CH_4_ production was observed from days 15 to 30 of incubation, reaching 16.74 ± 0.71 ml CH_4_ g^-1^ of VS (Figure 2).

**FIGURE 3:**
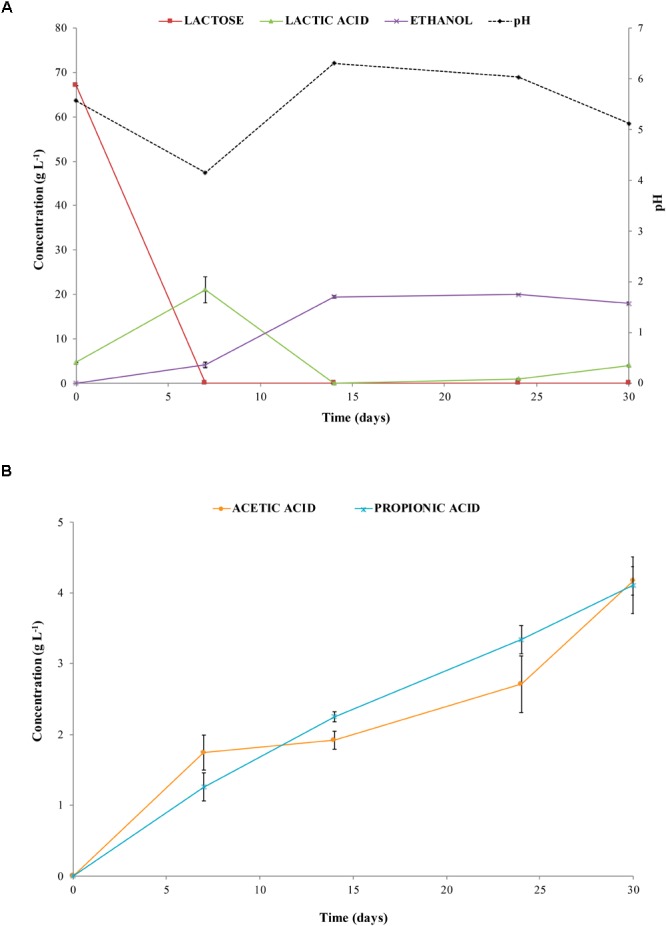
pH and concentration of lactose, lactic acid, and ethanol **(A)**, and concentration of acetic acid and propionic acid **(B)** in liquid samples collected from biodigesters at different times of incubation.

The amount of CO_2_ (% v/v) detected in the gas mixture ranged from 99 to 85% during the first week of incubation (at the beginning of the process) and from 9 to 12% when CH_4_ and H_2_ production occurred (data not shown). These results were in good agreement with trends of lactose and lactic acid concentration during incubation (Figure 3A). Actually, lactose was consumed and lactic acid concentration increased from 4.66 ± 0.11 g L^-1^ (T0) to 21.03 ± 2.11 g L^-1^ (T7) after 7 days of incubation. At day 14, lactic acid decreased until 0 g L^-1^ and an increase in the pH value up to 6.3 was observed decreasing to 5.1 at the end of the experiment. Whereas, ethanol concentration increased up to 19.69 ± 2.12 g L^-1^ after 14 days, remaining quite constant until day 30 (Figure 3A). At day 14, acetic and propionic acids concentrations were 1.92 ± 0.12 and 2.25 ± 0.07 g L^-1^, respectively, increasing up to 4.11 ± 0.5 g L^-1^ at the end of the process (Figure 3B).

### Microbial Analysis by Culture-Dependent Method

The monitoring of microbial viable counts during incubation time is listed in Table 2. The results showed that heterotrophic aerobic and anaerobic bacteria remained constant (approximately 7.2 and 7.4 log CFU mL^-1^, respectively) during the first week of incubation decreasing until 2.8–2.9 log CFU mL^-1^, at the end of the biodigestion process (*p* < 0.05). Similarly, aerobic spore-forming bacteria significantly decreased from 4.07 ± 0.09 log CFU mL^-1^ (T0) to 3.17 ± 0.07 log CFU mL^-1^ (T30). Whereas, an increase of 2 log was observed in anaerobic spore-forming bacteria and *Clostridia* loads from the initial time (3.91 ± 0.11 and 4.03 ± 0.36 log CFU mL^-1^, respectively) to day 14 of incubation (5.17 ± 0.36 and 5.95 ± 0.04 log CFU mL^-1^, respectively) decreasing again at the end of the incubation time (2.92 ± 0.05 and 3.77 ± 0.05 log CFU mL^-1^, respectively, *p* < 0.05). Finally, a high load of LAB was detected in the biodigesters until day 7 (6.6–7.6 log CFU mL^-1^), whereas at day 30 the load dropped showing a concentration of 1.00 ± 0.11 log CFU mL^-1^.

**Table 2:** Cultural monitoring of bacterial populations during incubation for 30 days at 30°C.

Microbial groups (log CFU mL^-1^)	Time			
	T0^∗^	T7^∗^	T14^∗^	T30^∗^
Heterotrophic aerobic bacteria	7.25 ± 0.14^A^	7.22 ± 0.18^A^	4.72 ± 0.67^B^	2.81 ± 0.07^C^
Heterotrophic anaerobic bacteria	7.30 ± 0.14^A^	7.36 ± 0.08^A^	4.51 ± 0.43^B^	2.88 ± 0.15^C^
Aerobic spore-forming bacteria	4.07 ± 0.09^A^	3.39 ± 0.08^B^	3.40 ± 0.30^B^	3.17 ± 0.07^B^
Anaerobic spore-forming bacteria	3.91 ± 0.11^B^	3.21 ± 0.09^C^	5.17 ± 0.36^A^	2.92 ± 0.05^C^
*Clostridia*	4.03 ± 0.36^C^	5.22 ± 0.36^B^	5.95 ± 0.04^A^	3.77 ± 0.05^C^
Lactic acid bacteria	6.60 ± 0.11^B^	7.58 ± 0.23^A^	4.47 ± 0.00^C^	1.00 ± 0.10^D^
Enterococci	3.26 ± 0.21^A^	3.30 ± 0.13^A^	1.00 ± 0.10^B^	1.00 ± 0.10^B^

*The values represent the means ± SD of three replicates. Different letters after the values indicate significant differences (*p* < 0.05). ^∗^T0, mixture of cheese whey and buttermilk inoculated at day 0 of incubation; T7, samples collected after 7 days of incubation; T14, samples collected after 14 days of incubation; T30, samples collected 1 after 30 days of incubation.*

### High-Throughput Sequencing of PCR-Amplified 16S rRNA Gene Sequences

#### Bacteria

A total of 677,030 high quality reads were analyzed for bacteria. The alpha-diversity was determined by calculating the Shannon diversity index based on OTUs of 97% identity (Table 3). The results revealed that the bacterial diversity significantly increased over time showing the highest Shannon diversity index in the biodigesters after 14 days (B1_T14 and B2_T14; *p* < 0.05) and 30 days of incubation (B1_T30 and B2_T30; *p* < 0.05). Good’s coverage indicated that 75–78% of the bacterial diversity was described in the samples.

**Table 3:** Observed diversity and estimated sample coverage for bacterial and archaeal 16S rRNA amplification from DNA extracted from biodigesters during the anaerobic process.

Sample^∗^	Bacteria			Archaea		
	No. OTUs	Shannon index^a^	Good’s coverage (%)	No. OTUs	Shannon index^a^	Good’s coverage (%)
Mix_T0	18211.40	6.39^D^	78.1	–	–	–
MixI_T0	20739.20	7.70^C^	75.5	6522.00	5.27^A^	87.7
B1_T7	20132.20	8.51^B^	77.1	6661.00	5.03^A^	87.4
B2_T7	20287.00	8.76^B^	76.8	6264.00	5.51^A^	88.4
B1_T14	21732.70	9.66^A^	76.3	4931.00	5.54^A^	90.9
B2_T14	22777.20	9.80^A^	74.9	6658.00	5.69^A^	87.5
B1_T30	22352.40	9.66^A^	75.2	6215.00	2.82^B^	88.0
B2_T30	21285.30	9.65^A^	76.8	6422.00	2.79^B^	87.6

*The entire data set was rarefied to 77670 reads or 47510 reads per sample, for bacteria and archaea respectively, before alpha-diversity was calculated. ^*a*^Different letters after Shannon index values indicate significant differences (*p* < 0.05). ^∗^Mix_T0, mixture of cheese whey and buttermilk at day 0 of incubation; MixI_T0, mixture of cheese whey and buttermilk inoculated at day 0 of incubation; B1_T7, sample collected from B1 after 7 days of incubation; B2_T7, sample collected from B2 after 7 days of incubation; B1_T14, sample collected from B1 after 14 days of incubation; B2_T14, sample collected from B2 after 14 days of incubation; B1_T30, sample collected from B1 after 30 days of incubation; B2_T30, sample collected from B2 after 30 days of incubation.*

**FIGURE 4:**
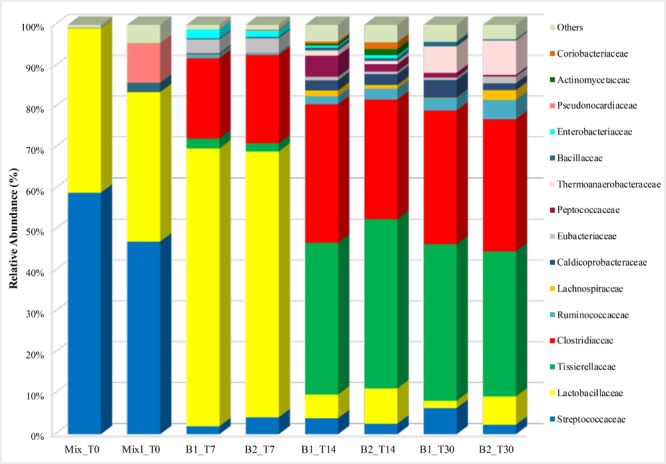
Abundance of bacterial families during the anaerobic process. Only OTUs with an incidence > 1% in at least one sample are shown. Mix_T0, mixture of cheese whey and buttermilk at day 0 of incubation; MixI_T0, mixture of cheese whey and buttermilk inoculated at day 0 of incubation; B1_T7, sample collected from B1 after 7 days of incubation; B2_T7, sample collected from B2 after 7 days of incubation; B1_T14, sample collected from B1 after 14 days of incubation; B2_T14, sample collected from B2 after 14 days of incubation; B1_T30, sample collected from B1 after 30 days of incubation; B2_T30, sample collected from B2 after 30 days of incubation.

The dynamics of bacterial populations were studied during the anaerobic process through their taxonomic composition detected at family level (Figure 4). Analysis of amplicon sequences showed *Streptococcaceae* (59% in Mix_T0 and 47% in MixI_T0) and *Lactobacillaceae* (40% in Mix_T0 and 36.5% in MixI_T0) families as dominant in the initial non-inoculated and inoculated mixture of cheese whey and buttermilk accounting for 99 and 83.5% of the total bacterial biodiversity in Mix_T0 and MixI_T0, respectively (Figure 4).

**FIGURE 5:**
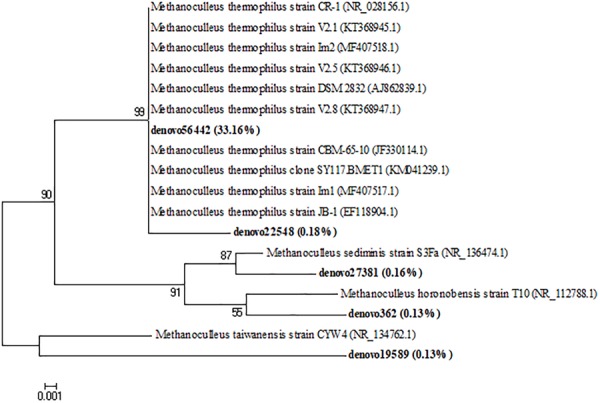
Neighbor-joining tree for the representative *Clostridiaceae* and *Tissierellaceae* OTUs (representatives with relative abundance > 0.1%). OTUs from this study were shown in bold reporting in brackets the total relative abundance. The sequence accession numbers of reference sequences from NCBI used for the phylogenetic analysis are shown in *parentheses* following the species name. Bootstrap values (>50%, expressed as percentages of 1,000 replications) are given at the nodes. The scale bar estimates the number of substitutions per site.

After 7 days of incubation, an increase of the abundance of *Lactobacillaceae* in the biodigesters B1 (67.8%) and B2 (64.9%) coupled with a *Streptococcaceae* decrease (1.9 and 4.1% in B1 and B2, respectively) was observed. Moreover, an increase of the *Clostridiaceae* family by 20% approximately, in both B1 and B2 was observed remaining quite stable until the end of the biodigestion process (around 32% in both B1_T30 and B2_T30, Figure 4). In addition to *Clostridiaceae, Tissierellaceae* was also a dominant family after 14 days (37.1 and 41.4% in B1 and B2, respectively) and 30 days of incubation (38.3 and 35.4% in B1 and B2, respectively). These two taxa accounted together for around the 70% of the bacterial biodiversity at the end of the anaerobic process (Figure 4). To investigate these two dominant bacterial families to a deeper level, a phylogenetic tree was constructed based on the values of OTUs with relative abundances higher than 0.1%. Phylogenetic analysis showed that the main *Clostridiaceae* OTUs, accounted for 2.15% of the total reads, were very similar to each other and were affiliated with *Clostridium thermopalmarium* species (Figure 5); while, other two OTUs were affiliated with *C. tyrobutyricum* and *C. clariflavum*. Regarding *Tissierellaceae* family, phylogenetic tree revealed that the main OTUs were affiliated with *Sporanaerobacter acetigenes* species, which accounted for 15.27% of the total reads (Figure 5). Among these, OTU denovo78367 and OTU denovo11901 were the most abundant which accounted for 7.35 and 3.63% of total reads, respectively.

Other bacterial taxa belonging to Clostridiales order, such as *Ruminococcaceae, Lachnospiraceae, Caldicoprobacteraceae, Eubacteriaceae* and *Peptococcaceae*, as well as several OTU identified as *Thermoanaerobacteraceae, Bacillaceae, Enterobacteriaceae, Pseudonocardiaceae, Actinomycetaceae*, and *Coriobacteriaceae* were also found to be lesser extent (Figure 4). For these taxa, the fluctuating relative abundance among samples does not allow to define a trend during incubation time.

**FIGURE 6:**
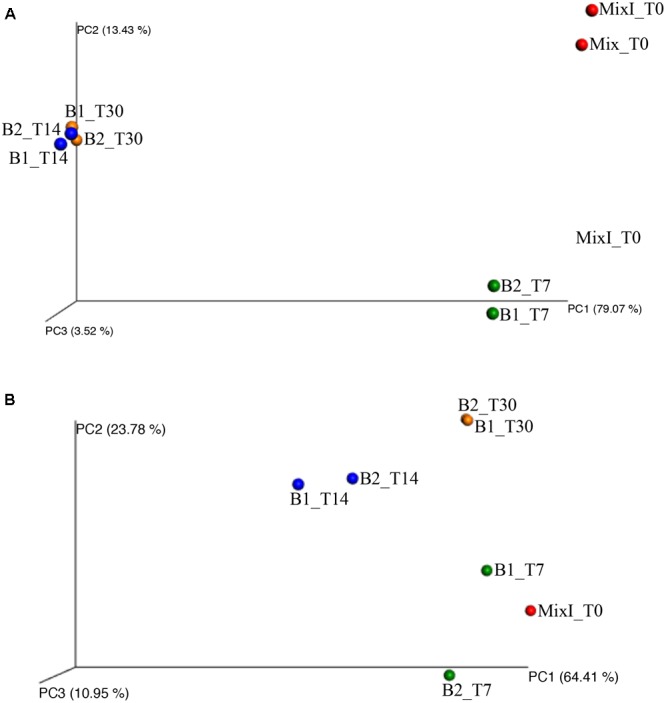
Principal coordinates analysis of weighted UniFrac distances for bacterial **(A)** and archaeal **(B)** 16S rRNA gene sequence data of samples during the incubation process. Color label. red: mixture of cheese whey and buttermilk at day 0 of incubation; green: samples collected after 7 days of incubation; blue: samples collected after 14 days of incubation; orange: samples collected after 30 days of incubation.

Besides, the principal coordinate analysis (PCoA) of the weighted UniFrac community distances revealed a marked difference between the microbiota in the early and final stages of the anaerobic process identifying three principal groups on the basis of the sampling time (Figure 6A). The four samples from biodigesters at days 14 and 30 of incubation (B1_T14, B2_T14, B1_T30, and B2_T30) clustered separately (Figure 6A). Moreover, the statistical ANOSIM test showed that the composition of bacterial community in the different samples during anaerobic process was significantly influenced by the sampling time (*p* < 0.01; *R* = 0.938).

#### Archaea

A total of 517,142 high quality reads were analyzed for archaea. The alpha-diversity was determined by calculating the Shannon diversity index based on OTUs of 97% identity (Table 3). Non-inoculated samples of cheese whey and buttermilk mixture (Mix_T0) was excluded from the analysis since very few archaeal reads were recovered (absolute abundance equal to 97 OTUs). The results showed that the archaeal diversity significantly increased after 5% of the inoculum was added and then strongly decreased at the end of the anaerobic process (Table 3; *p* < 0.05). Whereas, good’s coverage indicated that approximately 90% of the archaeal diversity was described in most of the samples.

**FIGURE 7:**
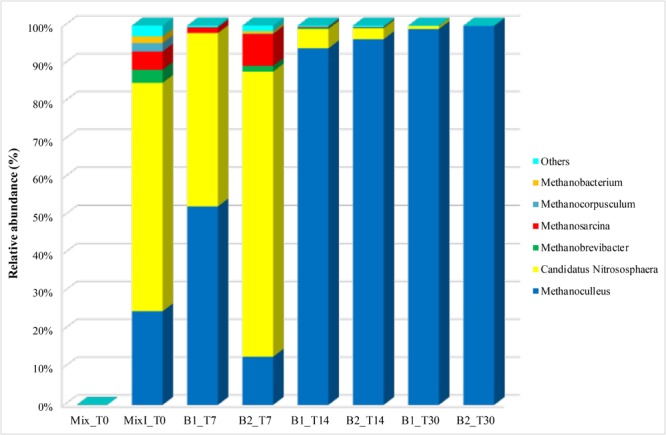
Abundance of archaeal genera during the anaerobic process. Only OTUs with an incidence > 1% in at least one sample are shown. Mix_T0, mixture of cheese whey and buttermilk at day 0 of incubation; MixI_T0, mixture of cheese whey and buttermilk inoculated at day 0 of incubation; B1_T7, sample collected from B1 after 7 days of incubation; B2_T7, sample collected from B2 after 7 days of incubation; B1_T14, sample collected from B1 after 14 days of incubation; B2_T14, sample collected from B2 after 14 days of incubation; B1_T30, sample collected from B1 after 30 days of incubation; B2_T30, sample collected from B2 after 30 days of incubation.

**FIGURE 8:**
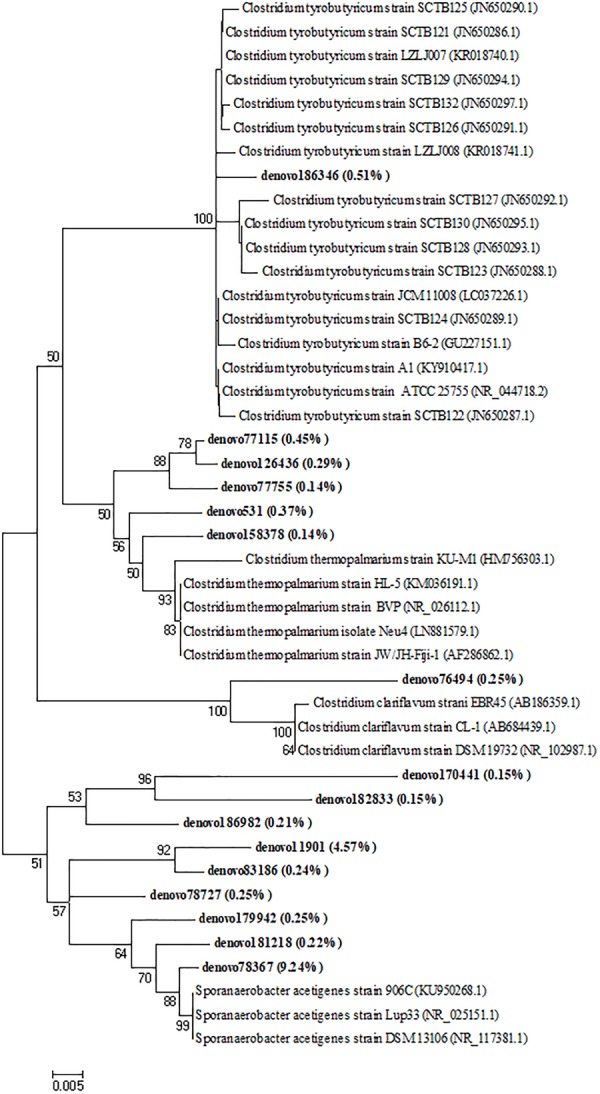
Neighbor-joining tree for the representative *Methanoculleus* OTUs (representatives with relative abundance > 0.1%). OTUs from this study were shown in bold reporting in brackets the total relative abundance. The sequence accession numbers of reference sequences from NCBI used for the phylogenetic analysis are shown in *parentheses* following the species name. Bootstrap values (>50%, expressed as percentages of 1,000 replications) are given at the nodes. The scale bar estimates the number of substitutions per site.

The archaeal taxa were examined at the genera level to determine the eventual occurrence of any significant shifts in their composition during the incubation time. Relevant results were shown from the samples collected at the initial time of the anaerobic process. Actually, marked differences were observed among inoculated and non-inoculated samples of cheese whey and buttermilk mixture where the relative abundance of archaeal genera was less than 0.1% (Mix_T0, Figure 7). Whereas, in the inoculated sample of the same mixture (MixI_T0, Figure 7), several archaeal genera were identified, such as *Methanoculleus* (24.7%), *Methanobrevibacte*r (3.4%), *Methanosarcina* (4.8%), *Methanocorpusculum* (2.2%), *Methanobacterium* (1.7%), and *Nitrososphaera* (60.2%). During incubation, non-methanogenic archaea belonging to the *Nitrososphaera* genus decreased from 5.1 to 2.9% (T14) to < 1% (T30). A similar trend was observed for the methanogenic archaea genera *Methanobrevibacte*r, *Methanosarcina, Methanocorpusculum* and *Methanobacterium*, which exhibited an abundance < 1% at the end of the incubation time. Otherwise, *Methanoculleus* was the dominant genus during all the process and its relative abundance increased up to 99.1–99.9% after 30 days of incubation (Figure 7). A phylogenetic tree based on the OTUs belonging to this genus showing the relative abundances higher than 0.1% was constructed, in order to evaluate which OTUs became dominant and who were their closest phylogenetic relatives. Phylogenetic analysis showed that the main *Methanoculleus* OTUs were affiliated with *M. thermophilus* (OTU denovo22548 and OTU denovo56442), *M. taiwanensis* (OTU denovo19589), *M. sediminis* (OTU denovo27381), *M. horonobensis* (OTU denovo362) and *Methanoculleus* spp. (OTU denovo26184), accounted for 33.83% of total reads (Figure 8). However, the dominant OTU was OTU denovo56442, strongly related to different strains of *M. thermophilus*, which explained most of the methanogenic biodiversity accounting for 32.32% of archaeal total reads (Figure 8).

The PCoA of the weighted UniFrac community distances showed a marked difference among the microbiota in the different samples over time. In fact, the sample of the initial inoculated mixture of cheese whey and buttermilk (MixI_T0) and those of the biodigesters at day 7 of incubation cluster together; while the two samples of the biodigesters at day 30 of incubation (B1_T30 and B2_T30) clustered separately (Figure 6B). Moreover, the statistical ANOSIM test showed that the composition of archaeal community in the different samples during anaerobic process was significantly influenced by the sampling time (*p* < 0.05; *R* = 0.729).

## Discussion

The microbial composition of the initial dairy waste mixture showed a high concentration of viable aerobic and anaerobic bacteria mainly belonging to LAB that commonly thrive in dairy waste (Kasmi et al., 2017). Since LAB produce lactic acid by homolactic and heterolactic fermentation processes (Palomba et al., 2012; Pradhan et al., 2017) they are well-adapted to the acidic environment (De Candia et al., 2007), typical of the cheese whey used in this study collected after fermentation and addition of organic acids during the production chain (Carvalho et al., 2013). Physical and chemical characteristics of the cheese whey are, actually, strictly related to the production chain, thus showing a wide range of organic matter concentration as reported in the literature (Ghaly and Singh, 1989; Ghaly and Kamal, 2004; Farizoglu et al., 2007; Saddoud et al., 2007; Azbar et al., 2009). In this study, cheese whey characteristics were in accordance with Ghaly and Singh (1989) that reported the concentrations of COD and VS equal to 75.8 and 47.9 g L^-1^, respectively. The high COD in cheese whey influenced the resulting COD in the mixture, indicating the potential of this substrate for feeding anaerobic process (Carvalho et al., 2013) and producing H_2_ and CH_4_. During incubation a relevant increase in hydrogen production was observed, simultaneously a decrease of the LAB concentration occurred until to reach a concentration less than 1 log CFU mL^-1^ at the end of the incubation time. The decrease in the LAB concentration was related to the increase in H_2_ production proving that lactic fermenters were the main competitors with the H_2_ producing microorganisms (Perna et al., 2013). This result was also confirmed by HTS for the bacterial 16S rRNA gene that showed a noticeable decrease (from 67% at day 7 to 6% at day 14) in the relative abundance of the *Lactobacillaceae* family when relevant H_2_ production occurred. Accordingly, it has been reported that *Lactobacillus* could not be related to high H_2_ production rate (Davila-Vazquez et al., 2009), although Yang et al. (2007) found higher abundance of *Lactobacillus* than *Clostridium* in anaerobic fermentation of cheese processing wastewater, thus reporting *Lactobacillus* related to H_2_ production.

On the other hand, in this work the increase in H_2_ production occurred simultaneously with an increase in *Clostridia* load. Actually, *Clostridia* have been reported to convert lactate into butyrate, CO_2_ and H_2_ in the presence of acetate (Barlow et al., 1991). Thus, the presence of these microorganisms could favor the production of hydrogen by fermentation of lactic acid followed by H_2_ production (Perna et al., 2013). For this reason, lactic acid was not detected at day 14 and an increase of acetic and propionic acids concentration was observed. This cultural approach allowed to acquire information about dynamics of viable bacterial populations which were able to live, grow, and die during the biodigestion process of this specific waste. Moreover, in order to obtain more information, a cultural-dependent approach was combined with a cultural-independent molecular method. According to Pandya et al. (2017) these techniques seem to be roughly equivalent and, when used in parallel, it is possible to obtain best results leading to major advances in the reliable knowledge of microbial populations living in an environment. The cultural results were confirmed by the HTS analysis that showed an increase in bacterial families belonging to Clostridiales order, such as *Tissierellaceae* and *Clostridiaceae*, which represented the most abundant bacterial taxa until the end of the incubation time. The selective pressure occurred in the ecosystem due to the presence of inoculum and the chemical–physical conditions of the anaerobic process selected these bacterial taxa, which are well-known to be involved in the H_2_ production (Navarro-Díaz et al., 2016; Alexandropoulou et al., 2018). In particular, in this study, the most dominant OTUs of *Clostridiaceae* and *Tissierellaceae* were affiliated with *Clostridium* spp. (*C. thermopalmarium, C. clariflavum*, and *C. tyrobutyricum*) and *Sporanaerobacter acetigenes*, respectively, which are commonly detected and isolated in many reactors for CH_4_ or H_2_ production (Hernandez-Eugenio et al., 2002; Shiratori et al., 2006; Jo et al., 2008; Weiss et al., 2008; Kim et al., 2010; Sasaki et al., 2012; Xia et al., 2014; Kumar et al., 2015; Cibis et al., 2016). The genus *Clostridium* comprised a large number of species that were often used to produce H_2_ (Jiang et al., 2013). Among them, *Clostridium tyrobutyricum* has been widely reported to be able to produce significant quantities of H_2_ from different sugars (Jiang et al., 2018) as well as it is also capable to utilize lactate as the main substrate for producing H_2_ (Wu et al., 2012; Noblecourt et al., 2018). Furthermore, fermentation products of *C. clariflavum* are H_2_, CO_2_, acetate, lactate, ethanol and a small amount of formate (Shiratori et al., 2009). Whereas *C. thermopalmarium* species are able to ferment sugars into butyric acid producing simultaneously H_2_, CO_2_, small amounts of acetate, lactate, and ethanol (Soh et al., 1991). Geng et al. (2010) demonstrated that the inoculation of *C. thermopalmarium* strain BVP (DSM 5974) increased biohydrogen production rather than the monoculture of *C. thermocellum* from cellulose.

In addition, Navarro-Díaz et al. (2016), reported the positive relationship between the increased H_2_ production with the presence of specific microbial families and genera, such as *Tissierellaceae* that may contribute also to complex substrate degradation because of its putative xylanolytic activity (Niu et al., 2009). *Sporanaerobacter acetigenes* strain Lup 33^T^, closely related to the OTU denovo78367, detected at the highest relative abundance, as well as related to the others representative OTUs affiliated to *Tissierellaceae*, was recognized as an acetogenic bacterium able to synthesize a mixture of VFAs, including acetate, isovalerate and isobutyrate, together with H_2_ and CO_2_ (Hernandez-Eugenio et al., 2002). Han et al. (2016) reported that *Sporanaerobacter acetigenes* was one of the main contributors for the hydrolysis and acidogenesis stages during anaerobic digestion of food waste-recycling wastewater (Han et al., 2016) as well as it was one of the primary species along with *Clostridium* during semi-continuous fermentation of *C. pyrenoidosa* biomass for H_2_ production (Xia et al., 2014). Moreover, the presence of *Ruminococcaceae* members in the samples taken at days 14 and 30 may be also correlated to H_2_ production since they are recognized as hydrogen producers and important substrate hydrolyzers (Niu et al., 2009).

In addition, ethanol was also produced in high amount starting from day 14 likely causing an inhibiting effect on hydrogen production (Hung et al., 2011) that therefore could have been higher than observed. This side effect can be related to the presence of *Streptococcaceae*, detected during incubation by using HTS for the bacterial 16S rRNA gene, since genera belonging to this family could produce ethanol thus inhibiting hydrogen production (Ren et al., 2007).

Furthermore, the environmental and physical conditions established in the system were also effective for the selection of the CH_4_ producing archaea. First, the archaeal populations’ presence in the biosystem was related to inoculum since the initial samples of the mixture of cheese whey and buttermilk had a relative abundance of archaea less than 0.1%. Secondly, the anaerobic environment in the biodigesters selected archaeal genera causing the decrease of the aerobic *Nitrososphaera* genus also detected in other studies of anaerobic digestion process (Li et al., 2014; Ventorino et al., 2018) even if it could be no able to produce methane (Chen et al., 2012).

During incubation, *Methanoculleus* genus percentage increased achieving very high relative abundance (99% at the end of the experiment) compared with that reported in other studies, such as Di Maria et al. (2017) (85%) and Leven et al. (2007) (18%), related to anaerobic digestion of organic waste. As reported by Poirier et al. (2016), *Methanoculleus* cooperate with acetate oxidizing bacteria belonging to the *Clostridiaceae* family detected in this study by both cultural and molecular approaches.

In agreement with Di Maria et al. (2017), *Methanoculleus* abundance increased during the incubation time and became dominant, whereas *Methanosarcina* decreased, as they are usually dominant in process fed with organic fraction of municipal solid waste (OFMSW) and activated sludge as inoculum (Lin et al., 2012). In this biosystem fed with a mixture of cheese whey and buttermilk, *Methanoculleus* was dominant using a hydrogenotrophic pathway to produce CH_4_ causing no organic acids consumption when methane production occurred. This result was in agreement with a previous study in which hydrogenotrophic pathway was identified as the main driver for CH_4_ production in batch reactors fed with dairy wastes, although, using different process conditions and a qualitative culture-independent method (DGGE) to microbial identification, *Methanobrevibacter* was found as the genus mostly related to the CH_4_ production (Pagliano et al., 2018). Interestingly, in this study, the dominant methanogenic archaeal OTU was affiliated with *M. thermophilus* which is able to produce CH_4_ from H_2_ or formate (Rivard and Smith, 1982). Recently, this species, with *M. beijingense*, was found to be dominant in a full-scale thermophilic anaerobic digester treating food wastewater (Lee et al., 2017). The other representative OTUs affiliated with *Methanoculleus* genus were closely related to *M. sediminis, M. taiwanensis*, and *M. horonobensis* previously isolated from sediments near a submarine mud volcano (Chen et al., 2015), deep-sea sediment (Shimizu et al., 2013) and deep subsurface groundwater from a diatomaceous shale formation (Weng et al., 2015), respectively.

Comparing the H_2_ and CH_4_ production with the literature, the production in this study (54.34 ± 0.15 L H_2_ kg VS^-1^ and 16.74 ± 0.71 L CH_4_ kg VS^-1^) were higher than that reported by Pagliano et al. (2018) (8.9 L H_2_ kg VS^-1^ and 2.2 L CH_4_ kg VS^-1^) using dairy waste as substrate and operating in batch mode. Different operating condition can promote the CH_4_ production, as reported by Lateef et al. (2014) that studied an anaerobic sequencing batch reactor (ASBR) fed with dairy waste achieving 35.6 L H_2_ Kg VS^-1^ and 627 L CH_4_ Kg VS^-1^.

Overall, obtained results highlighted that culture-dependent and independent approaches provided evidence for examining the relationship between bacterial and archaeal populations and biogas production in this biosystem. Besides, metagenomics sequencing technology is important to quantify the different microbial populations occurred in the reactors as well as to better understand the microbial dynamic during the anaerobic process of dairy wastes.

## Conclusion

Anaerobic biosystem was strictly influenced by microbial communities structure and dynamics derived from the inoculum, feedstock and the operating conditions. It represented a sustainable management process for the valorization of abundant wastes and by-products recovered from dairy industry. Polyphasic approach highlighted the function of specific bacterial populations that drove the biohydrogen production. Besides, the inoculation in the reactors with pelleted manure allowed Archaea development, revealing that methane was primarily formed through the hydrogenotrophic pathway, since *Methanoculleus* was the dominant genus during the process.

## Author Contributions

GP wrote the main manuscript text and performed the experiments. VV helped in writing the manuscript and performed the bioinformatics analysis. AP helped in physico-chemical analysis, in their interpretation and revised the manuscript. IR performed the microbial cultural analysis. FP revised the manuscript. OP conceived the study, participated in its design, and revised the manuscript.

## Conflict of Interest Statement

The authors declare that the research was conducted in the absence of any commercial or financial relationships that could be construed as a potential conflict of interest.
